# Advancing One Health Toxicology Through the Common Carp (*Cyprinus carpio*) Model

**DOI:** 10.3390/toxics14100857

**Published:** 2026-09-27

**Authors:** Maurizio Manera, Cosma Manera

**Affiliations:** 1 Department of Biosciences, Food and Environmental Technologies, University of Teramo, St. R. Balzarini 1, 64100 Teramo, Italy; 2 Independent Researcher, St. Antonio Di Vincenzo 12/2, 40129 Bologna, Italy; cosma.manera@gmail.com

**Keywords:** environmental biomonitoring, food safety, comparative pathobiology, contaminants of emerging concern, trophic transfer, biomarker indexing, xenobiotics

## Abstract

Translational toxicology faces a persistent model selection dilemma, as no single organism can serve as a universal surrogate. Within the holistic One Health paradigm, this review evaluates the common carp (*Cyprinus carpio* Linnaeus, 1758) as a versatile comparative model. A comparative evaluation of its somatic, ecological, and genomic attributes alongside benchmark teleosts like zebrafish, *Danio rerio* (Hamilton, 1822), highlights how the carp’s cosmopolitan distribution, benthivorous sediment-dwelling behaviour, and allotetraploid genome (Cs4R/4R-WGD) enable its survival in severely impacted aquatic environments precluded to less tolerant species, permitting continuous biomonitoring of environmental pollutants—among them heavy metals and emerging contaminants, including per- and polyfluoroalkyl substances (PFAS). A curated narrative synthesis reveals that while *C. carpio* is thoroughly established as an in situ environmental bioindicator (Sentinel Model) and an aquaculture cornerstone linking pollution to human dietary hazards (Consumer Link), its utility as a translational surrogate (Biomedical Surrogate) remains an emerging frontier. Grounded in evolutionary homology, its large somatic mass eliminates tissue pooling, enabling multi-organ pathobiological profiling and establishing multi-compartment systems, such as the kidney, as multipurpose biomarker organs. Ultimately, *C. carpio* offers unexploited potential to bridge laboratory mechanistic toxicology and planetary risk assessment.

## 1. Introduction. “Provando e Riprovando”: The Foundation of Modern Science

Systematic observation, hypothesis formulation, and experimental validation constitute the foundation of the modern scientific approach to research [1], dating back to the 17th century with the Florentine Accademia del Cimento original motto, “provando e riprovando” (trying and confuting) [2]. While the exact sciences construct simplified models to isolate physical variables [1], the biomedical sciences investigate complex living systems that cannot be reduced to simple mathematical, physical, or chemical formulations [1,3]. Researchers must therefore rely on surrogate organisms based on the evolutionary conservation of structures and functions [3,4]—a comparative methodology that introduces a persistent dilemma: identifying which species best models a target biological system to yield translationally valid knowledge [1,3,4].

In toxicology, this model selection challenge is increasingly framed within the One Health paradigm—a holistic framework recognizing that human, animal, and environmental health are deeply interconnected [5,6]. Within this paradigm, the utility of an animal model extends beyond traditional laboratory boundaries to inform human health through three distinct pathways [5,6]:

1. Indirect environmental risk assessment—the sentinel model: Because many animal species either occupy or overlap the same trophic levels and environmental niches as human populations, they function as vital in situ environmental sentinels, providing real-time diagnostic alerts of ecological degradation long before clinical endpoints manifest in human communities [5,6,7].
2. Direct dietary exposure risks—the consumer link: Bioindicator species that serve as commercial aquaculture or capture fishery commodities, accumulate persistent contaminants in edible tissues, directly linking aquatic ecotoxicology to food safety and human exposure risk assessment [5,8,9].
3. Translational comparative bioscience—the biomedical surrogate: Underpinned by “toxicity by descent” (phylotoxicology), conserved biochemical, endocrine, and immunological Adverse Outcome Pathways (AOPs) enable extrapolation of toxicokinetic and toxicodynamic mechanisms across vertebrate taxa [1,3,4,10].

Defining these foundational epistemological concepts is essential for contemporary toxicological research, as investigators too often treat animal models as uncritical “black boxes”, a myopic practice that ignores the conceptual boundaries of biological surrogacy and frequently leads to scientific misinterpretations and translational failures [1,3,11,12]. To navigate this comparative dilemma without lapsing into an exhaustive, encyclopedic survey of all bony fishes, this review deliberately focuses on the common carp (*Cyprinus carpio* Linnaeus, 1758) as a premier, non-standard teleost model within the One Health framework [13,14,15,16]. While acknowledging the historical and contemporary contributions of other established fish models (e.g., medaka, *Oryzias latipes* [Temminck & Schlegel, 1846], and fathead minnow, *Pimephales promelas* [Rafinesque, 1820]) [17,18,19], *C. carpio* is evaluated here in direct comparative contrast with the zebrafish, *Danio rerio* (Hamilton, 1822), the preeminent teleost benchmark in modern biomedical science [20,21,22,23,24]. By systematically examining its cosmopolitan distribution, somatic scale, allotetraploid genomic buffer, benthivorous ecology, physiological resilience, and multi-organ pathobiological utility, this review illustrates how the common carp bridges laboratory-based mechanistic toxicology with real-world environmental biomonitoring, human dietary risk, and cross-species hazard extrapolation as a translational model [8,13,14,15,16,25,26,27].

## 2. “The Best Material Model of a Cat Is Another, or Preferably the Same Cat”: Surrogacy and the Biomedical Model

The famous aphorism formulated by Rosenblueth and Wiener, “the best material model of a cat is another, or preferably the same cat” [28], highlights the central paradox of scientific modelling [1]. In living systems, this principle must be extended to “in the same time lapse”, as dynamic organisms continuously alter their biological state across life cycles, seasons, and environments, modifying their xenobiotic susceptibility [29,30,31,32]. By definition, a model is an intentional abstraction designed to replace complexity with a tractable construct [1]. Capturing every structural variable causes a model to inherit the target’s full complexity, defeating its experimental utility [28]. This representational paradox is dramatised in cartographic parables of the 1:1 scale map by Carroll and Borges—which coincided point-for-point with the territory but was ultimately abandoned as useless [33,34], collapsing under its own structural weight [33]. Epistemologically, this failure mirrors Baudrillard’s “precession of simulacra” [35], wherein over-standardised animal models construct a false “biomedical hyperreal” [35,36] that fails to reflect the chaotic genetic and environmental heterogeneity of natural or human populations [36]. Consequently, researchers must balance “models by analogy” against “models by homology” [1].

Attempting to evaluate an entire species through an isolated trait relies on traditional reductionism [37]. While disassembling organisms into independent components provides mechanistic details [37], reductionism ignores emergent properties and chaotic biological behaviour [38,39]. Because toxicological responses are non-linear, multi-scale perturbations governed by complex biological networks, they obey the systems biology maxim that “the whole is more than the sum of its parts” [38,40,41]. Thus, toxicological endpoints cannot be understood in isolation but must be evaluated as systemic perturbations of the whole living system [39,40].

Selecting appropriate surrogates within this framework is guided by the Krogh Heuristic [42,43]. Articulated by Krogh (1929) [42,43] and named by Krebs (1975) [11], the principle posits: “For a large number of problems there will be some animal of choice, or a few such animals, on which it can be most conveniently studied” [42,43,44]. This encourages the identification of “Krogh organisms” with specialised biological traits that render target mechanisms exceptionally accessible [42,43,44]. However, assuming a model optimal for one pathway represents a universal surrogate leads to fallacious generalisations [11,43]. This approach is balanced by the Wayne and Staves corollary: “No single organism (or technique) exists that can provide easy access to the diversity of hidden mechanisms that underlie all interesting and important physiological and biochemical problems” [12]. No single species serves as a universal surrogate; rather, researchers must deploy a suite of exemplary models to generate generalisable insights [45].

Historically, translational toxicology relied on mammalian analogues (e.g., rodents) as default surrogates for human biology [46]. Operating as “models by analogy”, this approach assumes similar functional patterns yield quantitative predictions [1]. However, mammalian analogues display poor predictivity for human systemic endpoints due to species-specific differences in absorption, distribution, metabolism, and excretion (ADME) pathways [46], as exemplified by historic failures like thalidomide teratogenicity [1,46]. Under the 3Rs (Replacement, Reduction, and Refinement) framework, regulatory agencies advocate replacing protected animals with New Approach Methodologies (NAMs) [38]. Yet, current cell-based or in silico NAMs cannot replicate whole-organism multi-organ physiology during chronic, low-dose exposures [47]. To bridge this gap, toxicology is shifting toward “models by homology” and “precision toxicology” grounded in “toxicity by descent” [4,10]. Because shared evolutionary history ensures that fundamental molecular targets, receptors, and Adverse Outcome Pathways (AOPs) are conserved across taxa [4,10], mapping homologous responses allows direct hazard extrapolation to human biology based on shared ancestry [4,10].

## 3. One Health: Beyond the Claim—Historical Foundations and Toxicological Reality

Though the “One Health” concept is frequently deployed as a novel, 21st-century paradigm—often functioning as an attractive institutional tagline or funding claim championed by international health organisations [48], stripping away modern institutional branding reveals that the “One Health” concept is far from being a recent innovation but rather the formalisation of an ancient holistic ethos [48,49]. Centuries before the term was co-opted as a policy slogan, veterinary scientists, physicians, and ecologists recognised that human, animal, and environmental health are indivisible aspects of a single biological continuum [49,50].

Actually, the historical bedrock of this integrated perspective was established in the 19th century by pioneering figures in comparative pathology [50,51,52]. In particular, Rudolf Virchow, the pre-eminent German physician and pathologist, famously asserted that “between animal and human medicine there is no dividing line—nor should there be” [50,53]. Furthermore, in his landmark treatise on cellular pathology (Die Cellularpathologie in ihrer Begründung auf physiologische und pathologische Gewebelehre), Virchow established the universal, conserved basis of pathology across living organisms, demonstrating that disease processes across all animal taxa are ultimately driven by structural and functional alterations at the cellular level [54,55]. This comparative philosophy was further propagated by other medical and veterinary researchers [51,52], culminating in the work of veterinary epidemiologist Calvin Schwabe [49,56]. Schwabe formally encapsulated this continuity under the term “One Medicine”, arguing that human and veterinary medicine share identical scientific foundations and must operate in unison against shared environmental and infectious threats [49,56]. Long before 21st-century administrative declarations, field veterinarians and ecologists were applying these principles daily to their professions, recognising ecosystems as complex networks where environmental disturbances inevitably cascade into biological pathology [48,49].

When rescued from policy rhetoric and restored to its empirical historical origins, the One Health framework provides toxicology with a multi-dimensional operational strategy [48,57]. Rather than treating environmental biomonitoring, food safety, and translational medicine as isolated domains, One Health unifies the three core concepts previously introduced in Section 1 by grounding them in a shared evolutionary and pathophysiological continuum [48,57].

First, the sentinel model gains deeper diagnostic power when interpreted through the lens of dynamic ecosystem exposure [57,58]. As established in Section 2, living organisms are non-static systems subject to temporal, seasonal, and physiological fluctuations [29,30,31,32]. Abiotic environmental sampling—such as static water or soil testing—fails to capture xenobiotic bioavailability, chemical mixtures, or bioaccumulation across time [59,60,61]. Biological sentinels occupying shared trophic levels integrate these complex exposure dynamics in situ [57,58]. Sublethal histological, cellular, and biochemical alterations in these organisms yield early diagnostic alerts, signalling indirect environmental hazards to human communities sharing the same ecosystem long before clinical pathologies manifest [57,58].

Second, this sentinel function directly intersects with human health through the dietary exposure route, transforming the bioindicator into a vital consumer link [62]. In aquatic ecosystems, many sentinel species are not merely laboratory abstractions but major commercial commodities within global wild-capture fisheries and aquaculture [25,62]. Lipophilic toxicants and persistent bioaccumulative chemicals—such as methylmercury, polychlorinated biphenyls (PCBs), and per- and polyfluoroalkyl substances (PFAS)—accumulate within edible muscle tissues and biomagnify up aquatic food chains [63,64]. Monitoring xenobiotic biotransformation and tissue residue dynamics in these species provides the empirical groundwork needed to establish maximum residue limits, manage food safety regulations, and protect public health from chronic dietary exposure [65,66].

Finally, the translational validity of these sentinel organisms is underpinned by the epistemological transition from “models by analogy” to “models by homology” detailed in Section 2 [1,4,10]. Grounded in Virchow’s cellular paradigm [54,55] and the evolutionary conservation of “toxicity by descent” [4], fundamental molecular pathways, receptor-ligand interactions, and cellular stress responses are deeply conserved across vertebrate lineages [4,10]. By mapping conserved AOPs in representative non-mammalian models, toxicologists can extrapolate mechanistic data directly to human pathophysiology [10,67,68]. This comparative synthesis elevates the sentinel organism from a passive environmental indicator to a predictive biomedical surrogate, unifying ecological health assessment with human toxicological risk management [3,57,67,68].

## 4. Beyond the Paraphyletic Misnomer: Teleostei as Comparative Models in Bioscience and Toxicology

From a cladistic perspective, the informal term “fish” does not denote a valid monophyletic clade but rather a non-monophyletic group of aquatic poikilotherm vertebrates [69,70,71]. In comparative bioscience, grouping these organisms together obscures a profound, counterintuitive evolutionary reality: a typical teleost fish (ray-finned fish) is phylogenetically closer to humans, sharing a more recent common ancestral node, than to a shark (cartilaginous fish), having diverged over 420 million years ago [69,70,71]. Although calling them collectively “fish” in lay discourse makes this proximity appear counterintuitive, establishing this cladistic framework is critical for rigorous comparative toxicological research [4,69].

Furthermore, teleosts must not be categorised using outdated, anthropocentric constructs such as “lower vertebrates” or “less evolved organisms” [72,73], going beyond the insidious anthropocentrism dating back to Aristotelian *scala naturae*, which falsely portrays evolution as a linear ladder progressing toward human perfection [72,73]. Modern “tree thinking” clarifies that evolution is a branching phylogenetic continuum [72,73]. No extant species derives directly from another living species; teleosts and tetrapods are sister lineages that have evolved independently along separate trajectories since their divergence from a common ancestor [69,72,73]. Similarly, no living species can be accurately described as “atavistic” or “archaic” as a whole; at most, a modern teleost may display ancestral (plesiomorphic) physiological traits alongside highly derived (apomorphic) evolutionary specialisations [72,73].

With over 30,000 described extant species, the infraclass Teleostei comprises more than 50% of all living vertebrate species—exceeding the combined species richness of all other vertebrate classes (amphibians, reptiles, birds, and mammals) combined [69,74]. This extraordinary evolutionary radiation, driven in part by the Teleost-Specific Whole Genome Duplication [(TS) WGD] [75], provides researchers with an immense diversity of physiological, metabolic, and genomic adaptations [75]. This vast biological diversity offers toxicologists unparalleled opportunities to select an ideal “Krogh model” tailored to a specific experimental context—balancing models by analogy and homology—rather than searching for an impossible universal surrogate [11,12,42,45].

Finally, teleosts serve as optimal candidate models for evaluating waterborne toxicants within the One Health paradigm [76,77,78]. Unlike terrestrial tetrapods, teleosts spend their entire life cycle fully immersed in water, maintaining an exceptionally intimate contact with the aquatic environment primarily via their branchial epithelium (gills) [79]. The gills represent a delicate, multifunctional, blood-perfused interface responsible for gas exchange, osmoregulation, ion transport, acid-base balance, and nitrogenous waste excretion [79]. This direct exposure makes the branchial tissue the primary target and real-time diagnostic sensor for waterborne xenobiotics [14,79,80]. Compared to aquatic invertebrates, teleosts share conserved vertebrate organ architecture, hepatic biotransformation machinery (e.g., CYP450 isoforms), endocrine axes, and AOPs, making experimental findings far more translationally transferable to mammals and human health [4,10,13,20,21,39,68,77]. Moreover, early developmental stages (such as the Fish Embryo Acute Toxicity test, OECD Test Guideline No. 236) preserve whole-organism multi-organ complexity while complying with international 3Rs bioethical frameworks [22,47].

## 5. Common Carp as a Candidate Model in Bioscience and the One Health Framework

While small laboratory teleosts such as the zebrafish, *Danio rerio* (Hamilton, 1822) have become dominant paradigms in high-throughput developmental genetics and translational pharmacology [20,21], the common carp offers unique biological, ecological, and somatic attributes that establish it as a premier candidate model within the integrated One Health framework [13,14]. Evaluating its utility requires a rigorous analysis of its historical background, ecological traits, genomic history, and a comparative assessment against established laboratory models.

Before evaluating *C. carpio* in detail, its role must be contextualised alongside other established teleost models in aquatic science. Historically, species such as the Japanese medaka, *O. latipes*; fathead minnow, *P. promelas*; goldfish, *Carassius auratus* (Linnaeus, 1758); and rainbow trout, *Oncorhynchus mykiss* (Walbaum, 1792), have served as fundamental workhorses in pure ecotoxicology and standardised regulatory hazard screening [81,82], as well as in specialised biomedical research models [17,18,19]. From a genomic standpoint, the lineage-specific fourth whole-genome duplication (Carp-specific 4th round of genome duplication, Cs4R/4R-WGD) events are not exclusive to *C. carpio*: the entire genus *Cyprinus* and the closely related genus *Carassius* share an ancestral cyprinid allotetraploidisation event occurring approximately 11–14 million years ago [75,83,84], while salmonids such as *O. mykiss* underwent an independent 4R-WGD event (Salmonid-specific 4th round of genome duplication, Ss4R/4R-WGD), approximately 80–100 million years ago) [75].

However, these established species face distinct ecological, or commercial constraints that limit their applicability across the full One Health framework:

1. Traditional ecotoxicological models (*O. latipes*, *P. promelas*): Predominantly deployed within single-species, pure ecotoxicological assays, these small teleosts lack the direct consumer/dietary food safety link—being non-commercial food commodities [17,18,19,81,82].
2. 4R-WGD cyprinid relatives (*Carassius* spp.): Although the entire genus *Carassius* shares the Cs4R genomic duplication [75], only *C. carpio* possesses the empirical evidence of a de facto cosmopolitan global distribution coupled with its status as one of the most heavily farmed and harvested commercial food fish in global aquaculture and inland fisheries [8,25].
3. 4R-WGD salmonid models (*O. mykiss*): Although rainbow trout share a 4R-duplicated genome and larger somatic scale, *O. mykiss* is a strict cold-water stenotherm requiring high dissolved oxygen levels and pristine aquatic environments [85,86,87]. Because anthropogenic industrial, municipal, and agricultural activities concentrate pollutants downstream in lowland valleys and lentic basins rather than mountain headwaters [88,89,90,91,92], rainbow trout cannot survive in warm, hypoxia-prone, heavily impacted lowland ecosystems where environmental toxicant burdens are most severe [85,86,87].

Conversely, in modern comparative bioscience, *D. rerio* stands as the undisputed reference benchmark for high-throughput genetics and mechanistic pathology [20,21,22,93,94]. Consequently, for the purposes of this review—which explicitly evaluates the applicability of the *C. carpio* model within the framework of One Health toxicology—*D. rerio* is intentionally selected as the primary comparative counterpart.

The common carp is a highly resilient, large-bodied cyprinid species with an ancient connection to human civilisation [8]. Native to the freshwater basins surrounding the Black, Caspian, and Aral Seas, *C. carpio* has been anthropogenically co-dispersed across all continents except Antarctica, achieving a truly cosmopolitan worldwide distribution in temperate and warm freshwater systems [8]. Historically, it stands as one of the earliest domesticated aquatic animals, with pond culture documented over 8000 years ago in Neolithic China and extensively developed during the Roman Empire [8]. Today, common carp remains a cornerstone of global inland aquaculture, contributing significantly to global fish production and serving as a major dietary protein source for human populations [8,25].

From an ecotoxicological perspective, carp is a benthivorous species that feeds by digging into organic sediments [95,96,97]. Because persistent organic pollutants (e.g., longer-chain PFAS, PCBs) and heavy metals bioaccumulate heavily in benthic sediments, the sediment-dwelling behaviour of carp exposes it directly to the most contaminated aquatic compartments [9,63,98,99]. Furthermore, its long lifespan (living 20–30 years) and large body mass (10–30 kg) allow it to function as a long-term temporal and spatial integrator of environmental bioaccumulation [9,16,100]. As an edible commercial commodity, toxicological biomonitoring in carp directly bridges environmental health with human food safety and dietary exposure risk assessment [9,101,102].

The genomic architecture of the common carp provides crucial insights into the physiological resilience that underpins its utility as an environmental sentinel. As previously stated, while all teleost fishes share an ancient (TS) WGD [75], *C. carpio* underwent a much more recent, lineage-specific allotetraploidisation event (Cs4R/4R-WGD) [75,83,84]. This 4R-WGD doubled its chromosome number to 2n = 100, contrasting sharply with diploid cyprinids such as *D. rerio*, which did not undergo a fourth WGD round and retained 2n = 50 chromosomes [75,83,84,103].

The retention of duplicated paralogous gene pairs (homoeologs) across the 4R carp genome provided extensive genetic raw material for evolutionary subfunctionalisation and neofunctionalisation [75,84,104]. Consequently, *C. carpio* exhibits massive expansions in cytoprotective, osmoregulatory, and biotransformation gene families, e.g., glutathione S-transferases (*GSTs*), cytochromes P450 (*CYPs*), and aquaporins (*AQPs*) [27,105,106,107].

This genetic redundancy acts as a powerful biochemical buffer [84,105,108]. The presence of functional homoeologs enables carp to maintain homeostasis under severe environmental stressors—such as severe hypoxia, thermal shifts, and heavy chemical contamination—where diploid species suffer metabolic collapse [27,108,109,110,111,112]. Although current evidence indicates that subgenome expression divergence in allotetraploid common carp provides genetic plasticity and tolerance during chronic pollutant exposure [27,113], further research is required to evaluate these mechanisms across broader toxicant classes, metabolic pathways, and comparative laboratory versus field settings. While this plasticity makes carp a remarkably hardy in situ sentinel, duplicated paralogous gene copies present challenges for transcriptomic biomonitoring [84,111,114,115]. Molecular assays (e.g., qPCR primers) must be designed with high specificity to distinguish between homoeologous transcripts, as uncritical primer design can result in non-specific gene copy amplification and confounded transcriptional biomarker profiles [114,116,117]. Beyond targeted PCR assays, high-throughput RNA sequencing (RNA-seq) encounters bioinformatic read-mapping ambiguities due to the high sequence identity (>90–95%) between retained homoeologous gene pairs across subgenomes A and B [83,84,118]. Furthermore, allotetraploid carp frequently display Homoeolog Expression Bias (HEB), wherein one subgenomic copy is dynamically regulated during pollutant exposure while the other remains silent or constitutively expressed; evaluating total combined transcript abundance uncritically can obscure true transcriptional mechanisms of toxicity [83,84,117]. Finally, because a single molecular target or biotransformation enzyme in diploid models often corresponds to multiple functional homoeologs in carp with divergent regulatory or catalytic properties, mapping Adverse Outcome Pathways (AOPs) requires evaluating multi-copy gene networks, complicating direct cross-species translational extrapolations [84,119,120].

A critical comparison between the common carp and the zebrafish reveals distinct operational advantages depending on whether the research context is high-throughput laboratory genetics or realistic One Health biomonitoring [9,13,16,20,21,22,93,94,97,101,102] (Table 1).

Importantly, evaluating *C. carpio* within One Health toxicology is not intended to position it as a faster or universally more sensitive toxicity screening tool than small laboratory teleosts. While embryonic and larval zebrafish excel in high-throughput, rapid mortality and developmental toxicity screening [20,21,22], hyper-sensitive assays often fail to mirror real-world ecological dynamics [141,142,143]. The operational utility of *C. carpio*, within the context of the present review, resides not in rapid acute feedback, but in its capacity to survive in severely impacted habitats—functioning as a long-term temporal integrator of bioaccumulation and enabling multi-organ pathobiological profiling on single individuals without tissue pooling [9,13,16,27,100].

While *D. rerio* is invaluable for developmental genetics due to its rapid generation time, optical transparency, sequenced diploid genome (2n = 50), and extensive transgenic strains [20,21,22,23], its utility as a wild environmental sentinel is limited [13,144]. Wild *D. rerio* is not a cosmopolitan species; its native distribution is strictly restricted to localised freshwater streams and rice paddies within the Indian subcontinent (spanning India, Pakistan, Bangladesh, Nepal, and Bhutan) [121,122]. Thus, zebrafish cannot serve as an in situ sentinel species for, e.g., European or American river basins [13,144].

Crucially, toxicology in zebrafish faces a major somatic bottleneck due to its minuscule adult body mass (0.5–1.0 g) [130,145]. Because individual organ tissues in zebrafish are tiny, toxicologists are routinely forced to pool organ tissue from multiple individuals to obtain sufficient analyte for a single assay [135,136,137,138]. This tissue pooling completely masks inter-individual variability and prevents the correlation of multi-organ pathological endpoints within a single exposed organism [135,136,137,138].

Conversely, the substantial body mass of *C. carpio* eliminates the tissue pooling requirement [13,133,134]. A single carp provides abundant tissue from multiple organ systems (liver, gills, brain, and kidney) [13,133,134]. This anatomical abundance enables multi-organ pathobiological profiling on a single individual, underpinning the concept of the carp kidney as a “multipurpose biomarker organ” [13]. Specifically, the carp kidney uniquely houses excretory nephrons, haematopoietic interstitium, and thyroid follicles within a single organ, allowing researchers to quantify and correlate nephrotoxic, immunotoxic, and thyrotoxic subindices simultaneously from a single tissue sample without pooling [13]. However, this substantial somatic scale introduces clear operational and logistical trade-offs. From a facility and husbandry perspective, while adult zebrafish can be maintained at high stocking densities in compact, fully automated benchtop recirculating rack systems with minimal spatial footprints, housing juvenile or adult carp requires large-capacity tanks and relatively higher water exchange volumes [8,21,96,122,130,140]. Furthermore, whole-body mount preparations are strictly restricted in *C. carpio* to early embryonic and small larval stages, whereas the small somatic size of *D. rerio* permits whole-body imaging and sectioning well into larval and juvenile stages [146,147,148,149,150].

Beyond somatic scale and husbandry footprints, the extended life history of *C. carpio* presents a classic trade-off between in situ biomonitoring utility and laboratory experimental feasibility. In natural wild environments and aquaculture settings, its long lifespan and delayed senescent profile represent major assets, enabling the carp to act as a long-term temporal integrator of chronic pollutant exposure, bioaccumulation kinetics, and progressive tissue pathobiology across multi-year exposure windows [9,16,98,100]. Furthermore, its large somatic mass permits longitudinal, non-lethal bio-sampling and clinical procedures from single identified individuals over extended holding periods [151,152,153]. Conversely, in controlled laboratory research, this protracted life cycle constitutes a severe operational drawback. Because *C. carpio* requires several years to achieve sexual maturation (typically 2–4 years for males and 3–5 years for females), conducting full life-cycle toxicity tests or multi-generational and transgenerational inheritance assays is operationally impractical [8,154,155]. In this regard, small laboratory teleosts with rapid generation times hold an undeniable advantage for high-throughput transgenerational genetics and reproductive hazard screening [154,155,156].

Recognising these life-history, physical, and infrastructural boundaries reinforces that *C. carpio* and *D. rerio* occupy complementary, fit for purpose roles along the comparative toxicological spectrum.

## 6. Delineating One Health Toxicology: Empirical Evidence and Case Studies in the Common Carp Model

Table 2 provides a curated narrative synthesis of representative research evaluating the common carp as an animal model within the One Health framework, whether the term “One Health” was explicitly stated by the authors or implicitly deduced from the study’s design, methodologies, and endpoints. To ensure an objective and targeted synthesis, representative literature was selected according to defined criteria focusing on *C. carpio* within ecotoxicology and biomonitoring frameworks. All candidate studies were subjected to critical evaluation to verify their inclusion in major international indexing databases (e.g., PubMed, Scopus), peer-reviewed status, methodological quality and experimental soundness (e.g., exposure realism, appropriate control groups, and validated analytical methods) and alignment with One Health principles, with no restrictions on publication date. Specifically, studies were included if they evaluated common carp exposure to environmental contaminants, in situ biomonitoring in anthropogenically impacted water bodies, or translational toxicological endpoints utilising environmentally relevant concentrations linked to human, animal, and ecosystem health—anchored by the Sentinel Model as the indispensable dimension. Conversely, papers focusing strictly on isolated ecotoxicological assays, routine aquaculture zootechnics, feed/nutrition optimisation, or infectious disease management lacking broader One Health implications were excluded. Although the term “One Health” is a relatively recent paradigm with institutional and funding implications—as critically discussed in Section 3, where the necessity to “go beyond the claim” was emphasised—it remains crucial from a practical perspective to encourage researchers to explicitly adopt this terminology. Doing so facilitates accurate indexing in literature repositories and fosters global awareness regarding the integration of the three core dimensions of One Health toxicology: indirect environmental risk assessment (the sentinel model), direct dietary exposure risks (the consumer link), and translational comparative bioscience (the biomedical surrogate).

To ensure complete methodological transparency, all percentage values reported in this section are presented solely for descriptive and informative purposes to illustrate structural trends within the 43 selected studies, rather than as statistical inferences or meta-analytic evidence about the entire published literature.

A critical evaluation of the 43 representative literature entries compiled in Table 2 reveals that only 5 out of 43 studies (~12%) explicitly incorporate the term “One Health” within their narrative. Notably, all 5 explicit studies originate from a single core experimental exposure fish cohort focused on sub-chronic perfluorooctanoic acid (PFOA) exposure, evaluating endpoints such as gill cytotoxicity, thyroid follicle segmentation and degeneration, kidney biomarker subindices, and glomerular filtration barrier integrity [13,14,178,179,180]. The vast majority of the literature—38 out of 43 studies (~88%)—only implicitly addresses the One Health paradigm through ecotoxicological, biomonitoring, and food safety endpoints without explicit conceptual branding. Furthermore, a holistic integration spanning all three One Health dimensions simultaneously is achieved in only 6 out of 43 studies (~14%), including key investigations on PFOA bioconcentration and depuration dynamics [177], branchial mitochondria-rich cell cytotoxicity [14], integrated renal biomarker indexing [13], nasal inflammatory pathway alteration [188], metabolic and reproductive gene expression profiling [181], and rodlet cell degranulation texture analysis [182]. Regarding the distribution of individual dimensions across the compiled literature (Table 3), the Sentinel Model (indirect environmental risk assessment) is ubiquitous, appearing in all the studies (100%), as a consequence of inclusion criteria. The Consumer Link (direct dietary exposure risk) follows closely, featured in 40 out of 43 (~93%) of the publications, highlighting the carp’s prominent dual role as an in situ bioindicator and an edible commercial aquaculture commodity. Finally, the Biomedical Surrogate dimension (translational comparative bioscience) is represented in 9 out of 43 (~21%) of the studies, underscoring the growing utility of teleost tissue-level pathobiology for cross-species hazard extrapolation.

From an epistemological perspective, it is crucial to recognise that these three One Health dimensions do not represent rigid, physically mutually exclusive variables. Rather, they constitute fluid, overlapping conceptual frameworks designed to capture the intrinsic multidimensionality of the One Health paradigm [5,6,38,48,56,57]. Within this framework, the Sentinel Model serves as the foundational anchor—the indispensable “sine qua non”—without which toxicological inquiry loses its environmental grounding and ceases to embody true One Health principles [5,6,57,58]. Consequently, the ubiquitous representation of the Sentinel Model dimension across the compiled literature acts as a conceptual positive control, confirming alignment with One Health inclusion criteria. The remaining dimensions—the Consumer Link and Biomedical Surrogate—do not operate in isolation but rather function as complementary, additive layers built upon this primary ecotoxicological foundation [9,10,62,68]. Notably, this clear thematic imbalance—wherein the Sentinel Model accounts for 100%, the Consumer Link for ~93.0%, and the Biomedical Surrogate for only ~21%, with full three-dimension integration achieved in just ~14.0% of the reviewed literature—does not indicate an intrinsic limitation of the model organism itself. Rather, it highlights that the full operationalisation of *C. carpio* within the One Health triad remains an emerging paradigm. Current research predominantly exploits the carp for environmental biomonitoring and food safety, while its potential as a translational, tissue-level biomedical surrogate remains significantly under-explored. By evaluating *C. carpio* at the confluence of these overlapping domains, toxicologists can trace a continuous continuum from in situ ecosystem degradation to human dietary risk and conserved pathophysiological mechanisms [1,3,4,5,6,10,13,14,39,62].

Furthermore, a critical explorative methodological evaluation of the selected corpus (Table 3) illustrates how experimental approaches directly align with the specific One Health dimensions under investigation. In research predominantly focused on environmental biomonitoring (the Sentinel Model) and dietary risk assessment (the Consumer Link), studies rely primarily on purely analytical and descriptive methods (~42% and ~45% of their respective cohorts). For these dimensions, the principal scientific objective is to confirm toxicant exposure, quantify tissue bioaccumulation, and calculate regulatory hazard quotients, while underlying subcellular mechanisms often remain unexpressed or secondary [196,197]. Conversely, where the translational surrogate approach is evident (the Biomedical Surrogate), mechanistic methodologies become prevalent (~33% purely mechanistic) or co-exist alongside analytical chemistry (~67% overlapping approach). In this translational context, research extends beyond merely detecting an analyte to systematically document and elucidate its pathobiological effects across cellular, tissue, and multi-organ systems [197,198]. Crucially, this methodological pattern matches the structural distribution observed across Table 2: complete three-dimension One Health convergence (~14%) is achieved exclusively by studies employing an overlapping approach that bridges rigorous exposure analytics with mechanistic pathobiology. Rather than indicating a biological limitation of *C. carpio*, this distribution demonstrates that full One Health integration requires demanding, multi-disciplinary experimental protocols—explaining why translational biomedical profiling remains an under-exploited frontier in the current literature.

Expanding the comprehensive evaluation across the compiled entries in Table 2 provides critical insight into their experimental paradigms and environmental relevance. Accounting for possible overlapping, 25 out of 43 studies (~58%) involve laboratory-based or mesocosm experimental exposures, whereas 17 out of 43 studies (~40%) assess wild field biomonitoring or in situ environmental monitoring. When classified into mutually exclusive study designs, the literature comprises purely experimental exposures (23 studies; ~53%), purely natural wild environments (12 studies; ~28%), comparative designs spanning natural wild sites and commercial aquaculture ponds (4 studies; ~9%), purely aquaculture ponds (1 study; ~2%), integrated rice–fish culture systems (1 study; ~2%), experimental exposures linked with aquaculture pond settings (1 study; ~2%), and mixed designs combining wild capture with subsequent clean-water experimental depuration and restocking (1 study; ~2%).

Crucially, aquaculture ponds and integrated agro-ecosystems represent pivotal socio-ecological interfaces where environmental pollution, aquatic animal health, and human dietary risk intersect [8,16,96,101]. In total, 6 out of 43 studies (~14%) incorporate aquaculture ponds into their experimental or observational frameworks. These artificial and semi-natural impoundments function as key transitional zones: unlike pristine wild habitats or artificial laboratory tanks, aquaculture ponds continuously receive agricultural runoff, sediment-bound pollutants, and anthropogenic chemicals while serving as primary production sites for commercial human food fish [8,96,101]. Similarly, integrated rice–fish culture systems exemplify this dual sentinel–consumer nexus, wherein carp are simultaneously exposed to agricultural xenobiotics (such as pesticides, herbicides, and fungicides) within flooded water-sediment matrices and evaluated for bioaccumulation in edible tissues destined for human consumption [16].

A significant finding revealed by this setting analysis concerns the dosing philosophy adopted across experimental studies. All the 25 studies incorporating an experimental exposure setting strictly account for environmentally relevant concentrations of the target analytes. Rather than subjecting organisms to supra-environmental or acute lethal concentrations typically employed in traditional regulatory hazard screening, experimental research on *C. carpio* overwhelmingly focuses on chronic, low-dose exposures that mirror real-world aquatic contamination levels—such as ambient surface water and sediment concentrations of per- and polyfluoroalkyl substances (PFAS), pharmaceuticals, veterinary antibiotics, and heavy metals [13,14,16,27,99,112]. This strict adherence to environmentally realistic exposure regimes ensures that observed ultrastructural, histopathological, and transcriptomic alterations yield translationally valid diagnostic indicators, successfully bridging the gap between laboratory-based mechanistic toxicology and real-world ecological risk management within the One Health paradigm [13,14,16,27,112].

A detailed analysis of the analyte categories investigated across the study entries in Table 2 provides a clear map of how toxicological research (within the One Health framework) on *C. carpio* is currently partitioned between legacy pollutants and contaminants of emerging concern (CECs). The researched chemical stressors can be grouped into six primary toxicological classes, though partial overlapping should be considered: heavy metals & metalloids (20 studies; ~47%), per- and polyfluoroalkyl substances (PFAS) (9 studies; ~21%), pharmaceuticals & personal care products (PPCPs) (9 studies; ~21%), endocrine disrupting chemicals (EDCs) (2 studies; ~5%), pesticides (2 studies; ~5%), and microplastics (1 study; ~2%).

Heavy metals and metalloids (spanning Pb, Cd, Cr, Zn, Cu, As, Hg, Ni, Fe, Mn, Co, and Sb) continue to command the largest share of carp ecotoxicological literature [9,100,101,159,167,173,175]. Because metals are non-biodegradable, persistent, and readily bioaccumulated by benthivorous organisms, these studies are heavily anchored in wild-field biomonitoring across natural lakes, rivers, and reservoirs, as well as commercial aquaculture ponds [9,100,101,159,167,173,175]. By evaluating tissue residue kinetics in edible muscle alongside internal organ pathology, heavy metal research in carp directly bridges in situ sentinel biomonitoring with human dietary exposure risk assessments and public health food safety standards (the Consumer Link) [9,100,101,159,167,173,175].

Conversely, modern ecotoxicological research on *C. carpio* has heavily pivoted toward contaminants of emerging concern [13,14,99,187,188], which collectively account for ~42% of the reviewed literature. Within this modern domain, PFAS (~21%) and Pharmaceuticals/PPCPs (~21%) are investigated with equal prominence.

PFAS/PFOA research represents a highly specialised, experimentally controlled body of work [13,14,133,177,178,179,180,181,182]. These studies leverage, among others, advanced transmission electron microscopy (TEM), digital texture analysis, and morphometrics to decipher sub-cellular organelle degeneration, branchial mitochondria-rich cell cytotoxicity, and endocrine-thyroidal disruption under environmentally realistic exposure regimes [13,14,178,179,180,182].

Research in pharmaceuticals & PPCPs addresses the chronic ecological pressures of municipal wastewater effluents and agricultural runoff across diverse active ingredients, including antibiotics, psychiatric drugs, NSAIDs, and beta-blockers [183,184,185,186,187,188,189,190,191]. Rather than isolated pathobiological observations, these independent studies utilise *C. carpio* to evaluate multi-systemic endpoints—ranging from biotransformation kinetics and Phase I/II xenobiotic enzyme induction to intestinal microbiota dysbiosis, gut mucosal barrier disruption, and neuro-olfactory inflammatory alterations [183,184,185,186,187,188,189,190,191]. This body of work demonstrates the carp’s capacity to model the systemic, ecological, and translational hazards of emerging contaminants within the One Health paradigm.

Finally, under-represented contaminant classes—including EDCs (~5%), pesticides (~5%), and microplastics (~2%)—constitute a combined ~12% of the compiled dataset. Despite their lower relative representation, these studies address critical exposure vectors. For instance, pesticide evaluations investigate xenobiotic bioaccumulation and oxidative stress dynamics in carp reared within integrated agro-ecosystems such as rice–fish culture systems [16]. Similarly, microplastic research highlights a novel One Health transmission vector: the bioaccumulation of synthetic polymer fragments (e.g., polypropylene, polystyrene, polyethylene terephthalate, and rayon) in edible carp muscle transferred directly via contaminated commercial fish meal and aquafeeds [195]. This demonstrates that human dietary exposure risks can originate directly from agricultural feed supply chains, reinforcing the necessity of an integrated One Health approach [195].

## 7. Conclusions

The persistent comparative dilemma of translational science confirms that no unique, universal animal model can serve as a perfect surrogate for all biological systems [1,3]. As underscored by the Wayne and Staves corollary, no single species provides a master key to the vast diversity of physiological and toxicological mechanisms [12]. Experimental models must therefore be selected dynamically and fit for purpose, guided by the Krogh heuristic to align with the specific research question, environmental setting, and exposure scenario [11,42,43,44].

Within the modern One Health framework, the common carp offers a highly versatile combination of biological, ecological, and evolutionary attributes [8,13,14]. Genomically, its lineage-specific allotetraploidisation event (Cs4R/4R-WGD) created a rich genetic buffer of duplicated homoeologous gene pairs [75,83,84], underpinning expansions in cytoprotective, osmoregulatory, and xenobiotic biotransformation gene families (such as *GSTs*, *CYPs*, and *AQPs*) [27,105,106,107]. This genetic plasticity grants *C. carpio* exceptional physiological resilience, enabling it to maintain homeostasis under severe hypoxia, thermal shifts, and heavy chemical contamination across deeply impacted lotic and lentic ecosystems [27,108,109,110,111,112].

Ecologically and geographically, *C. carpio* is de facto cosmopolitan across temperate and warm freshwater worldwide [8,9], acting as a ubiquitous in situ environmental sentinel (the Sentinel Model) across diverse river basins and reservoirs where geographically restricted laboratory models like zebrafish cannot be deployed [8,13,21,97]. Furthermore, its benthivorous, sediment-dwelling feeding habits and long lifespan (20–40 years) render it an exceptional temporal and spatial integrator of persistent, bioaccumulative, and hydrophobic aquatic pollutants [9,16,95,96,97,98,99,100].

Crucially, as an edible and economically vital cornerstone of global freshwater aquaculture, toxicological research in carp directly bridges environmental degradation with human food safety and public health, providing key empirical data to manage dietary exposure pathways (the Consumer Link) [9,16,25,101,102]. Simultaneously, its large somatic mass permits extensive multi-organ pathobiological profiling on single individuals without tissue pooling, establishing the carp kidney and other tissues as highly effective multipurpose biomarker systems [13,100,101,102,133].

Finally, because fundamental biochemical, endocrine, and immunological pathways are deeply conserved across vertebrate lineages, toxicological mechanisms mapped in this teleost yield translationally valid insights applicable to other species, including humans (the Biomedical Surrogate) [1,3,4,10,67,68]. Nevertheless, as revealed by the present literature evaluation, full integration across all three One Health dimensions remains an emerging frontier rather than a fully realised norm, with its capacity as a translational Biomedical Surrogate still largely under-explored. By transitioning from reductionist “models by analogy” to evolutionary “models by homology” under the One Health paradigm, *C. carpio* rises above its historical role as a passive environmental bioindicator, serving as a powerful, predictive surrogate to monitor, model, and mitigate planetary chemical hazards [4,5,10,13,48,57].

## Figures and Tables

**Table 1:** Comparative evaluation of common carp (*Cyprinus carpio*) and zebrafish **(***Danio rerio*) as experimental models and bioindicators in One Health toxicology.

Trait/Parameter	Common Carp	Zebrafish	Reference
Primary Scientific Role	Ecological sentinel, in situ bioindicator, multi-organ systemic model, and food safety model.	High-throughput laboratory model, genetics, developmental biology, and drug screening.	[8,9,13,16,20,21,22,93,94,101,102]
Native Geographic Distribution	Eurasia (Ponto-Caspian basins); anthropogenically introduced globally (cosmopolitan wild distribution).	Restricted strictly to the Indian subcontinent (India, Pakistan, Bangladesh, Nepal, Bhutan).	[8,121,122]
Environmental Resistance/Resilience	Exceptional; highly tolerant to hypoxia, thermal fluctuations, and heavy pollution.	Moderate; requires standardized, highly controlled laboratory water conditions.	[26,122,123,124,125,126]
Trophic Relevance	High; sediment-dwelling benthivore, bioaccumulating pollutants from sediments; edible food fish.	Negligible; pelagic micro-predator in localized streams; not consumed by humans.	[100,121,127,128,15]
Adult Body Mass & Dimensions	Large; adult mass typically 1.0–20 kg; total length up to 1.0 m.	Minuscule; adult mass 0.5–1.0 g; total length 3.0–4.0 cm.	[129,130]
Lifespan	Long; 20–40 years, enabling multi-year, chronic bioaccumulation studies.	Short; 3.0–4.0 years in captivity; highly restricted temporal integration.	[129,131,132]
Tissue Pooling Requirement	Absent; large somatic mass permits extensive multi-endpoint sampling from a single individual.	Mandatory; minuscule organs require pooling tissue from multiple fish, masking variability.	[13,133,134,135,136,137,138]
Genome & Ploidy	Allotetraploid (2n = 100); experienced a recent lineage-specific 4R-WGD (~11–14 Mya).	Diploid (2n = 50); experienced only the ancient teleost-specific 3R-WGD (~320 Mya).	[75,83,84,103,139]
Xenobiotic-related Gene Redundancy	High; expanded duplicated detoxifying gene families (GSTs, AQPs, CYPs).	Standard; single-copy or 3R-retained duplicates; lower genetic buffer capacity.	[27,105,106,107]
Laboratory Husbandry	Requires larger tanks/mesocosms; generation time ~1–2 years to maturity.	Highly optimized; low space requirements, rapid generation time (~3 months), high fecundity.	[8,21,96,122,130,140]
Transgenic & Genetic Lines	Limited; genomic manipulation is complex due to 4R tetraploid genome and long life cycle.	Abundant; extensive collections of transgenic, mutant, and reporter lines.	[23,84,108,114]

Abbreviation Keys: AQPs, Aquaporins; CYPs, Cytochromes P450; 3R, Third-round genome duplication; 3R-WGD, Third-round whole-genome duplication; 4R, Fourth-round genome duplication; 4R-WGD, Fourth-round whole-genome duplication; GSTs, Glutathione S-transferases; Mya, Million years ago.

**Table 2:** Representative literature synthesis evaluating toxicological studies on the common carp (*Cyprinus carpio*) model within the One Health framework, categorized by target organ, analytical methodology, contaminant class, exposure setting, and One Health dimension.

Key Biomarker/Target Organ	Methodologies Used	Analyte Categories	Study Setting & Environmental Relevance	Main Outcomes	One Health Concept & Dimension	Reference
Muscle	Flame Atomic Absorption Spectrophotometry (FAAS), THQ, CR methods	Heavy Metals (Fe, Cu, Mn, Zn, Cd, Cr, Pb)	Natural Environment	Mn, Cd, Pb, and Cr exceeded permissible limits for safe consumption; Lake Hayqe showed higher levels than Lake Tana; Cd and Cr posed acceptable levels of carcinogenic risk.	Implicitly Deduced (Sentinel Model, Consumer Link)	[157]
Muscle, Liver, Kidney, Heart, Gill, Gill Bone	Inductively Coupled Plasma Mass Spectrometry (ICP-MS)	Potentially Toxic Elements (PTEs) (As, Cd, Co, Cr, Cu, Fe, Hg, Mn, Ni, Pb, Zn)	Natural Environment	Bioaccumulation of As, Cd, Pb, and Zn exceeded human consumption limits in mining-impacted site. Kidney is the primary target for As and Cd; Liver for Cu, Fe, and Zn.	Implicitly Deduced (Sentinel Model, Consumer Link)	[158]
Muscle, Gills, Liver, Kidney, Intestine	Atomic Absorption Spectrophotometry, Diagnostic Kits (CAT, SOD, MDA), ELISA (Cytokines), Light Microscopy	Heavy Metals (Cr, Cd, Pb)	Experimental Setting (Environmentally Relevant)	Bioaccumulation order: Gills > Liver > Kidney > Intestine > Muscle; significant oxidative stress and immunotoxic cytokine elevation; severe gill lamellar fusion and hepatic vacuolization.	Implicitly Deduced (Sentinel Model, Consumer Link)	[159]
Liver, Kidneys, Gills	ICP-MS, Spectrophotometry (GST, Glycogen, Protein)	Toxic Metals (Zn, Cu, Pb, Cd)	Natural Environment & Aquaculture Ponds	Significant spatial/seasonal bioaccumulation (Zn > Cu > Pb > Cd). GST induction and glycogen depletion indicate high metabolic costs for detoxification.	Implicitly Deduced (Sentinel Model, Consumer Link)	[160]
Liver, Spleen, Head-kidney, Thymus, Blood	AAS, Enzymatic assays (ALT, AST, ALP, CK), ELISA (IgM), Light Microscopy, RT-qPCR (MT, GPX)	Heavy Metals (Cd, Ni, Cu, Cr)	Natural Environment & Aquaculture Pond	Highest accumulation in liver and head-kidney; increased hepatic/renal markers; tissue necrosis; upregulation of MT and GPX genes; elevated serum IgM.	Implicitly Deduced (Sentinel Model, Consumer Link)	[161]
Kidney	Light Microscopy, TEM, qRT-PCR, Western blotting, CCK-8 assay, Immunofluorescence	Arsenic and Zinc	Experimental Setting (Environmentally Relevant)	Arsenic induced renal ferroptosis and lipid peroxidation via YAP-TFR pathway; Zinc supplementation alleviated effects by inhibiting YAP activation.	Implicitly Deduced (Sentinel Model, Consumer Link)	[162]
Muscle	Flame AAS	Heavy Metals (Cd, Cr, Pb, Zn)	Natural Environment	Cr levels exceeded FAO permissible limits; Zn and Cd were within limits; Pb was not detected. BAF indicates significant transfer from water.	Implicitly Deduced (Consumer Link, Sentinel Model)	[163]
Gills, Liver, Muscle, Blood	AAS, Hematology (RBC/WBC/Hb), Spectrophotometry	Heavy Metals (Pb)	Experimental Setting (Environmentally Relevant)	Highest bioaccumulation in gills; reduction in RBC/WBC/Hb; increased hepatic enzymes and glucose; behavioral changes and scale erosion observed.	Implicitly Deduced (Sentinel Model, Consumer Link)	[164]
Muscle	ICP-OES, BAF calculation, Human Health Risk Assessment (THQ, HI, CR)	Heavy Metals (Fe, Zn, Cu, Cr, Ni, Pb, Cd)	Natural Environment	Pb, Cd, and Zn exceeded safe limits. High BAF for Zn and Cr. Carcinogenic risks for Cr^6+^ and Pb^2+^ exceeded safety thresholds.	Implicitly Deduced (Sentinel Model, Consumer Link)	[165]
Gills, Liver, Muscle, Skin	ICP-OES	Trace Metals (Mn, Pb, Zn, Hg, Cr)	Natural Environment	Metal concentration order: Gills > Liver > Skin > Muscles. Mn, Hg, and Cr exceeded WHO/FEPA standards for human consumption.	Implicitly Deduced (Sentinel Model, Consumer Link)	[166]
Liver, Gonads	ICP-OES, Biometric Indices (CF, GSI, HSI)	Essential and non-essential elements (Na, Mg, K, Fe, Zn, Ba, Cr, Mn, Ni, Se, Ag, Cd, Sb, Pb, Cu, As)	Natural Environment	Essential elements (Mg, Na, K) dominated. Liver showed higher accumulation than gonads. Size-dependent bioaccumulation pathways identified for Na, Mg, K, Mn, and Cu.	Implicitly Deduced (Sentinel Model, Consumer Link)	[167]
Kidney, Gills, Gut, Muscle, Skin, Scales	ICP-OES, Light microscopy, PCR, 16S rRNA sequencing, Biochemical assays (ROS, SOD, MDA, TP)	Heavy Metals (Cr, Cd, Cu mixture)	Experimental Setting (Environmentally Relevant)	High mortality at the highest concentration; gill lamellae fusion and glomerular necrosis; increased MDA; significant gut microbial dysbiosis (Firmicutes/Proteobacteria shifts).	Implicitly Deduced (Sentinel Model, Consumer Link)	[168]
Gut, Liver, Muscle, Blood Serum	RT-qPCR (MT, GPX), Light Microscopy, AAS, Biochemical Assays	Heavy Metals (Cd, Ni, Cu, Cr)	Natural Environment vs. Aquaculture Pond	Metal accumulation exceeded WHO/FAO standards in muscle; histopathological alterations in gut and liver; significant overexpression of MT and GPX genes.	Implicitly Deduced (Sentinel Model, Consumer Link)	[169]
Liver, Gills, Muscle, Intestine	ICP-MS, THQ determination	Heavy Metals (Zn, Cu, As, Pb, Cd, Hg)	Natural Environment	Highest Cu/As in liver, Zn/Pb in intestine, Hg in muscle. Total THQ > 1 indicates health risk for local residents, primarily driven by Arsenic.	Implicitly Deduced (Sentinel Model, Consumer Link)	[170]
Intestine (Microbiota)	High-throughput sequencing (16S rRNA)	Heavy Metals (Cadmium)	Experimental Setting (Environmentally Relevant)	Cd exposure restructured microbial communities, reducing beneficial *Cetobacterium* and increasing Cd-resistant and pathogenic bacteria.	Implicitly Deduced (Sentinel Model, Consumer Link)	[171]
Muscle, Gills, Liver, Kidney, Gonad	ICP-OES	Heavy Metals (Cd, Cr, Cu, Zn, Fe, Pb, Mn, Hg, Ni, Se)	Natural Environment	Fe and Zn accumulation highest; Cd, Ni, Hg, Pb, and Mn in muscle exceeded permissible national/international health limits.	Implicitly Deduced (Sentinel Model, Consumer Link)	[172]
Muscle	Graphite Furnace AAS, CVAAS, Health Risk Assessment	Heavy Metals (Zn, Pb, Cd, Hg)	Aquaculture pond	All heavy metals in muscle were below FAO/WHO limits; muscle did not act as a bioaccumulative reservoir (BCF < 1000); low carcinogenic risk.	Implicitly Deduced (Sentinel Model, Consumer Link)	[173]
Liver, Gills, Muscle	Flame AAS, Light microscopy	Heavy Metals (As, Pb, Hg)	Natural Environment and Aquaculture Pond	Mercury exceeded WHO limits in liver (60%), gills (100%), and muscle (90%). Microscopy showed telangiectasia and liver necrosis.	Implicitly Deduced (Sentinel Model, Consumer Link)	[174]
Blood, Intestine, Skin, Liver, Gills, Muscle	Comet Assay, AAS	Heavy Metals (Zn, Ni, Cr, Cu, Cd, Pb, Fe, Mn, Hg)	Natural Environment	Genotoxicity sequence: blood > intestine > skin > liver > gills > muscle. Mercury exceeded permissible limits in water and fish tissue.	Implicitly Deduced (Sentinel Model, Consumer Link)	[175]
Blood (Plasma/Whole)	Biochemical assays (GSH, CAT, SOD, MDA)	Wastewater (Heavy Metals and Nutrients)	Natural Environment	Significant increase in CAT and MDA; decrease in SOD and GSH in polluted site, indicating high oxidative stress and lipid peroxidation.	Implicitly Deduced (Sentinel Model, Consumer Link)	[176]
Gallbladder, Kidney, Liver, Brain, Gills, Gonads, Intestine, Muscle, Skin, Scales	LC-MS/MS	PFAS (PFOA and metabolites: PFBA, PFPeA, PFHxA, PFHpA)	Experimental Setting (Environmentally Relevant)	PFOA bioaccumulated in all organs (highest in gallbladder/kidneys). Four biotransformation products identified; PFBA dominant. Changes in HSI and GSI confirm endocrine disruption.	Implicitly Deduced (Sentinel Model, Consumer Link, Biomedical Surrogate)	[177]
Gills	Transmission Electron Microscopy (TEM)	PFAS (Perfluorooctanoic acid—PFOA)	Experimental Setting (Environmentally Relevant)	Concentration-dependent cytotoxic changes: mitochondrial degeneration, Golgi and endoplasmic reticulum stress, autophagic activation, and necrosis at high doses.	Explicitly Stated (Sentinel Model, Consumer Link, Biomedical Surrogate)	[14]
Kidney (Renal Thyroid Follicles)	Light Microscopy, Morphometry, Image Analysis	PFAS (Perfluorooctanoic acid—PFOA)	Experimental Setting (Environmentally Relevant)	PFOA exposure induced de novo folliculogenesis. Significant reductions in colloid perimeter and area observed at environmental relevant concentrations.	Explicitly Stated (Sentinel Model, Biomedical Surrogate)	[178]
Kidney, Thyroid Follicles	Light Microscopy, TEM, Morphometry, Z-scoring	PFAS (Perfluorooctanoic Acid—PFOA)	Experimental Setting (Environmentally Relevant)	Induced glomerular hyperfiltration, podocyte effacement (nephrotoxic), rodlet cell degranulation (immunotoxic), and thyroid degeneration (thyrotoxic).	Explicitly Stated (Sentinel Model, Consumer Link, Biomedical Surrogate)	[13]
Kidney (Glomerular Filtration Barrier)	TEM	PFAS (Perfluorooctanoic acid—PFOA)	Experimental Setting (Environmentally Relevant)	Ultrastructural alterations: podocyte effacement, pedicel fusion, and basement membrane disarrangement, indicating glomerulonephrosis.	Explicitly Stated (Sentinel Model, Biomedical Surrogate)	[179]
Liver, Gonads, Blood, Gills, Muscle, Kidney, Brain	HPLC-ESI-MS/MS, Light Microscopy, IHC (Anti-PCNA), Biometric Indices	PFAS (Perfluorooctanoic acid—PFOA)	Experimental Setting (Environmentally Relevant)	Significant accumulation in blood and liver; atretic oocytes and suppressed spermatogenesis at the highest concentration; increased hepatocyte proliferation.	Implicitly Deduced (Sentinel Model, Consumer Link)	[133]
Thyroid Follicles	Light/Electron Microscopy, HPLC-ESI-MS/MS, Morphometry	PFAS (Perfluorooctanoic Acid—PFOA)	Experimental Setting (Environmentally Relevant)	Increased follicle number and vesiculation; RER enlargement and cytoplasm vacuolation indicate impaired thyroglobulin production and degeneration.	Explicitly Stated (Sentinel Model, Biomedical Surrogate)	[180]
Liver, Gonads, Blood	LC-MS, HPLC-ESI-MS/MS, RT-qPCR	PFAS (Perfluorooctanoic acid—PFOA)	Experimental Setting (Environmentally Relevant)	PFOA bioaccumulated at the highest concentration. CYP19A expression altered in male and female gonads at both concentrations, indicating endocrine disruption without bioaccumulation.	Implicitly Deduced (Sentinel Model, Consumer Link, Biomedical Surrogate)	[181]
Kidney/Rodlet Cells	Light Microscopy, Texture Analysis, LDA, Gray-level Co-occurrence Matrix	PFAS (Perfluorooctanoic acid—PFOA)	Experimental Setting (Environmentally Relevant)	Induced significant rodlet cell degranulation.	Implicitly Deduced (Sentinel Model, Consumer Link, Biomedical Surrogate)	[182]
Brain, Gills, Liver, and Kidney	HPLC-EC-UV, MS/MS, Nitrocefin test, FEIA, Spectrophotometry (LPX, HPC, PCC, SOD, CAT, GPx)	Antibiotics: Amoxicillin (AMX) and Amoxicilloic acid (AMA)	Experimental Setting (Environmentally Relevant)	AMX degrades to AMA via beta-lactamases. AMA induces oxidative stress, with the kidney most susceptible.	Implicitly Deduced (Sentinel Model, Consumer Link)	[183]
Liver, Intestine, Muscle	EROD/BFCOD activity, qPCR, Western blotting, GC-FID, 16S rDNA amplicon	Pharmaceuticals and Personal Care Products (PPCPs)	Experimental Setting (Environmentally Relevant)	Induction of CYP1A and CYP3A-like activities; shift in muscle fatty acid composition (lower n-3/n-6 ratio); and significant alteration of intestinal microbiome.	Implicitly Deduced (Sentinel Model, Consumer Link)	[184]
Liver, Kidney, Intestine, Gills	LC-HRMS, Spectrophotometry	CECs, PhACs (Antidepressants, Beta-blockers, NSAIDs), Nutrients	Natural Environment/Experimental Setting (Environmentally Relevant)	Clean water restocking eliminates PhACs within 14 days, but metabolic profiles remain altered for >60 days; polluted water triggers rapid antioxidant response.	Implicitly Deduced (Sentinel Model, Consumer Link)	[185]
Liver, Gills, Brain, Muscle	HPLC, Light Microscopy, Spectrofluorometry (DCFH-DA), Morphometry	Antibiotics (Sulfamethoxazole)	Experimental Setting (Environmentally Relevant)	Dose-dependent ROS production in brain and gills; histopathological changes include bile stagnation and branchial lamellar fusion; low muscle bioconcentration factors.	Implicitly Deduced (Sentinel Model, Consumer Link)	[186]
Blood Plasma	LC/ESI-MS/MS, Isotope dilution, Solid phase extraction	PPCPs (Antidepressants, Antibiotics, Stimulants)	Natural Environment	Bioaccumulation of 13 PPCPs; oxazepam showed the highest BAFP. Antidepressant levels in plasma were near human therapeutic levels.	Implicitly Deduced (Sentinel Model, Consumer Link)	[187]
Olfactory epithelium	Light microscopy, qPCR, Biochemical assays, Network toxicology	Antibiotics (Norfloxacin)	Experimental Setting (Environmentally Relevant)	Impairment of immune homeostasis; hemorrhagic lesions and goblet cell hyperplasia in olfactory epithelium; oxidative stress via NF-κB pathways.	Implicitly Deduced (Sentinel Model, Consumer Link, Biomedical Surrogate)	[188]
Intestine, Muscle	Light Microscopy, Biochemical Assays, RNA-Seq, qRT-PCR, THQ	Antibiotics (Norfloxacin)	Experimental Setting (Environmentally Relevant)	Reduced intestinal villi height; induction of oxidative stress; impaired innate immune barrier; activation of NF-kB; THQ < 1 for muscle consumption.	Implicitly Deduced (Sentinel Model, Consumer Link)	[189]
Liver, Blood Plasma	Transcriptomics (RNA-seq), Biochemical Assays, Light Microscopy, qRT-PCR, HPLC-MS	Antibiotics (Norfloxacin)	Experimental Setting (Environmentally Relevant)	Long-term exposure induced hepatotoxicity and disordered glucose/lipid metabolism; triggered pathways related to nonalcoholic fatty liver disease (NAFLD).	Implicitly Deduced (Sentinel Model, Consumer Link)	[190]
Gut (Microbiota)	Metagenomic sequencing, Alpha/Beta diversity, CARD database	Antibiotics (Norfloxacin)	Experimental Setting (Environmentally Relevant)	Chronic exposure (100 ng/L) reduced microbial alpha diversity and inhibited metabolism pathways; increased abundance of ARGs (parE and gyrA).	Implicitly Deduced (Sentinel Model, Consumer Link)	[191]
Muscle, Liver, Kidney, Gill, Blood	HPLC, LC-MS/MS, Pharmacokinetic analysis, Acute toxicity test	Benzoylurea insecticides (Diflubenzuron)	Aquaculture pond & Experimental Setting (Environmentally Relevant)	Bioaccumulated in lipid-rich tissues (Kidney > Liver > Muscle). Peak muscle levels 6-fold water concentration. Dietary risk identified for young children.	Implicitly Deduced (Sentinel Model, Consumer Link)	[192]
Brain, Gills, Liver, Muscle	HPLC, GC-MS/MS, Spectrophotometry, TBARS	Pesticides (Insecticides and Fungicides)	Rice–fish culture system	Inhibition of brain AChE; increased lipid peroxidation in liver, gills, and muscle; increased GST and CAT activity; NPSH depletion indicating stress.	Implicitly Deduced (Sentinel Model, Consumer Link)	[16]
Muscle, Liver, Gills, Gonad	GC-MS, Risk Assessment (Monte-Carlo simulation)	Phenols (BPA, 4-n-NP), Estrogens (E1, DES, EE2)	Natural Environment	High BPA levels in gonads suggest reproductive threats; liver/muscle ratios >1 confirm chronic exposure to phenols and natural estrogens.	Implicitly Deduced (Sentinel Model, Consumer Link)	[193]
Muscle, Liver, Testis, Brain, Gills	LC50 Bioassay, Behavioral Monitoring, UV-VIS Spectrometry, ELISA, GC-MS	Phthalates (Diethyl Phthalate—DEP)	Experimental Setting (Environmentally Relevant)	DEP exposure caused vitellogenin induction in males, decreased GSI, increased HSI, and altered metabolic enzyme activities (AST, ALT).	Implicitly Deduced (Sentinel Model, Consumer Link)	[194]
Gastrointestinal tract and Gills	FTIR Spectroscopy and Microscopy	Microplastics (PP, PS, PE, PET, Rayon)	Experimental Setting (Environmentally Relevant)	Positive correlation between microplastics in fish meal and tissue bioaccumulation; 72% of uptake in GI tract vs. 28% in gills.	Implicitly Deduced (Sentinel Model, Consumer Link)	[195]

Abbreviation Keys: 4-n-NP, 4-n-Nonylphenol; 16S rDNA, 16S Ribosomal DNA; 16S rRNA, 16S Ribosomal RNA; AAS, Atomic absorption spectrometry; AChE, Acetylcholinesterase; ALP, Alkaline phosphatase; ALT, Alanine aminotransferase; AMA, Amoxicilloic acid; AMX, Amoxicillin; ARGs, Antibiotic resistance genes; AST, Aspartate aminotransferase; BAF, Bioaccumulation factor; BAFP, Plasma bioaccumulation factor; BCF, Bioconcentration factor; BFCOD, Benzyloxyresorufin-O-debenzylase activity; BPA, Bisphenol A; CARD, Comprehensive antibiotic resistance database; CAT, Catalase; CCK-8, Cell Counting Kit-8; CECs, Contaminants of emerging concern; CF, Fulton’s condition factor; CK, Creatine kinase; CR, Carcinogenic risk; CVAAS, Cold vapour atomic absorption spectrometry; CYP1A, Cytochrome P450 1A; CYP19A, cytochrome P450 family 19 subfamily A; CYP3A, Cytochrome P450 family 3 subfamily A; DCFH-DA, 2′,7′-dichlorofluorescin diacetate; DEP, Diethyl phthalate; DES, Diethylstilbestrol; E1, Estrone; EE2, 17α-Ethinylestradiol; ELISA, Enzyme-linked immunosorbent assay; EROD, Ethoxyresorufin-O-deethylase activity; FAAS, Flame atomic absorption spectrometry; FEIA, Fluorescent enzyme immunoassay; FAO, Food and Agriculture Organization; FEPA, Federal environmental protection agency; FTIR, Fourier-transform infrared spectroscopy; GC-FID, Gas chromatography-flame ionisation detection; GC-MS, Gas chromatography-mass spectrometry; GC-MS/MS, Gas chromatography-tandem mass spectrometry; GI, Gastrointestinal; GPx/GPX, Glutathione peroxidase; GSH, Reduced glutathione; GSI, Gonadosomatic index; GST, Glutathione S-transferase; Hb, Haemoglobin; HI, Hazard index; HPC, Hydroperoxide content; HPLC, High-performance liquid chromatography; HPLC-EC-UV, High-performance liquid chromatography with electrochemical and ultraviolet detection; HPLC-ESI-MS/MS, High-performance liquid chromatography–electrospray ionization–tandem mass spectrometry; HPLC-MS, High-performance liquid chromatography–mass spectrometry; HSI, Hepatosomatic index; ICP-MS, Inductively coupled plasma mass spectrometry; ICP-OES, Inductively coupled plasma optical emission spectrometry; IHC, Immunohistochemistry; IgM, Immunoglobulin M; LC50, Median lethal concentration; LC-HRMS, Liquid chromatography-high-resolution mass spectrometry; LC-MS, Liquid chromatography-mass spectrometry; LC-MS/MS, Liquid chromatography-tandem mass spectrometry; LC/ESI-MS/MS, Liquid chromatography/electrospray ionisation-tandem mass spectrometry; LDA, Linear discriminant analysis; -LPX, Lipid peroxidation; MDA, Malondialdehyde; MS/MS, Tandem mass spectrometry; MT, Metallothionein; NAFLD, non-alcoholic fatty liver disease; NF-κB, Nuclear factor kappa-light-chain-enhancer of activated B cells; NPSH, Non-protein sulfhydryls; NSAIDs, Non-steroidal anti-inflammatory drugs; PCC, Protein carbonyl content; PCNA, Proliferating cell nuclear antigen; PE, Polyethylene; PET, Polyethylene terephthalate; PFAS, Per- and polyfluoroalkyl substances; PFBA, Perfluorobutanoic acid; PFHpA, Perfluoroheptanoic acid; PFHxA, Perfluorohexanoic acid; PFOA, Perfluorooctanoic acid; PFPeA, Perfluoropentanoic acid; PhACs, Pharmaceutically active compounds; PP, Polypropylene; PPCPs, Pharmaceuticals and personal care products; PS, Polystyrene; PTEs, Potentially toxic elements; PCR, polymerase chain reaction; qPCR/qRT-PCR, Quantitative real-time polymerase chain reaction; RBC, Red blood cell; RER, Rough endoplasmic reticulum; RNA-seq, RNA sequencing; ROS, Reactive oxygen species; RT-qPCR, Reverse transcription-quantitative polymerase chain reaction; SOD, Superoxide dismutase; TBARS, Thiobarbituric acid reactive substances; TEM, Transmission electron microscopy; TFR, Transferrin receptor; THQ, Target hazard quotient; TP, Total protein; UV-VIS, Ultraviolet-visible spectroscopy; WBC, White blood cell; WHO, World health organisation; YAP, Yes-associated protein.

**Table 3:** Methodological classification (purely analytical/descriptive, purely mechanistic, and overlapping) of curated Cyprinus carpio research across individual One Health dimensions, two-dimension partial convergence, and full three-dimension convergence (N = 43).

One Health Dimension	Total Studies (n)	% of Total Dataset (N = 43)	Purely Analytical (n, % of Cohort)	Purely Mechanistic (n, % of Cohort)	Overlapping Both (n, % of Cohort)
Individual Dimensions					
Sentinel Model	43	100%	18 (~42%)	9 (~21%)	16 (~37%)
Consumer Link	40	~93%	18 (~45%)	6 (~15%)	16 (~40%)
Biomedical Surrogate	9	~21%	0 (0%)	3 (~33%)	6 (~67%)
Two-Dimension Partial Convergence					
Total Two-Dimension Convergence (Exact 2 Dimensions)	37	~86%	18 (~49%)	9 (~24%)	10 (~27%)
-Sentinel + Consumer Link (only)	34	~79%	18 (~53%)	6 (~18%)	10 (~29%)
-Sentinel + Biomedical Surrogate (only)	3	~7%	0 (0%)	3 (100%)	0 (0%)
-Consumer Link + Biomedical Surrogate (only)	0	0%	0 (0%)	0 (0%)	0 (0%)
Three-Dimension Full Convergence					
Full 3-Dimension Convergence (Sentinel + Consumer + Biomedical)	6	~14%	0 (0%)	0 (0%)	6 (100%)

Footnote: All percentage values reported in this table are presented solely for descriptive and informative purposes to illustrate structural trends within the 43 selected studies, rather than as statistical inferences or meta-analytic evidence about the entire published literature. Percentages in parentheses preceded by the tilde symbol (~) reflect approximated intra-cohort proportions (relative share within that specific row’s cohort). Purely Analytical studies comprise analytical chemistry residue quantification and/or descriptive pathology; Purely Mechanistic studies evaluate subcellular, biochemical, or molecular pathways without concurrent tissue residue analytics; Overlapping studies integrate exposure chemistry with mechanistic pathobiology on the same cohort. The 37 two-dimension partial convergence studies combined with the 6 three-dimension full convergence studies account for the entire dataset (37 + 6 = 43 studies; 100%).

## Data Availability

No new data were created or analysed in this study. Data sharing is not applicable to this article.

## Abbreviations

The following abbreviations are used in this manuscript:

3Rs	Replacement, Reduction, and Refinement
4R-WGD	Fourth-Round Whole Genome Duplication
ADME	Absorption, Distribution, Metabolism, and Excretion
AOPs	Adverse Outcome Pathways
AQPs	Aquaporins
CECs	Contaminants of Emerging Concern
Cs4R	Carp-specific 4th round of genome duplication
CYP450	Cytochromes P450
CYPs	Cytochromes P450
EDCs	Endocrine-Disrupting Chemicals
GSTs	Glutathione S-transferases
HEB	Homoeolog Expression Bias
NAMs	New Approach Methodologies
NSAIDs	Non-Steroidal Anti-Inflammatory Drugs
OECD	Organisation for Economic Co-operation and Development
PCBs	Polychlorinated Biphenyls
PCR	Polymerase Chain Reaction
PFAS	Per- and Polyfluoroalkyl Substances
PFOA	Perfluorooctanoic Acid
PPCPs	Pharmaceuticals and Personal Care Products
qPCR	Quantitative Real-Time Polymerase Chain Reaction
RNA-seq	RNA Sequencing
Ss4R	Salmonid-specific 4th round of genome duplication
TEM	Transmission Electron Microscopy
(TS) WGD	Teleost-Specific Whole Genome Duplication
WGD	Whole Genome Duplication
